# Caregiver Status and Diet Quality in Community-Dwelling Adults

**DOI:** 10.3390/nu13061803

**Published:** 2021-05-26

**Authors:** Sharmin Hossain, May A. Beydoun, Michele K. Evans, Alan B. Zonderman, Marie F. Kuczmarski

**Affiliations:** 1 Laboratory of Epidemiology and Population Sciences, Intramural Research Program, National Institute on Aging, Baltimore, MD 21224, USA; baydounm@mail.nih.gov (M.A.B.); me42v@nih.gov (M.K.E.); zondermana@mail.nih.gov (A.B.Z.); 2 Department of Behavioral Health and Nutrition, University of Delaware, Newark, DE 19716, USA; mfk@udel.edu

**Keywords:** caregiver, diet quality, African American, HEI-2010, health disparities, elderly

## Abstract

Objective: We investigated cross-sectional and longitudinal associations of diet quality with middle-aged caregiver status. Methods: Caregiving in the Healthy Aging in Neighborhoods of Diversity across the Life Span (HANDLS) study (57.7% women, 62% African American (AA)) was measured at waves 3 (2009–2013) and 4 (2013–2017) (mean follow-up time 4.1 years). Diet quality was assessed by the Healthy Eating Index 2010 (HEI-2010) derived from two separate 24 h diet recalls. Multivariable ordinary least square regression was performed for cross-sectional analyses of the association of wave 4 caregiving with wave 4 HEI-2010. Wave 3 caregiving was examined both cross-sectionally and with annual rate of change in HEI using mixed-effects linear regression Models. Multivariable models were adjusted for age, sex, and poverty status. Results: Cross-sectional analyses at wave 4 demonstrate an inverse association of frequent caregiving (“Daily or Weekly” vs. “Never”) for grandchildren with HEI-2010 total score (i.e., lower diet quality) among Whites (β = −2.83 ± 1.19, *p* = 0.03, Model 2) and AAs (β = −1.84 ± 0.79, *p* = 0.02,). The “cross-sectional” analysis pertaining to grandchildren caregiving frequency suggested that frequent caregiving (i.e., “Daily or Weekly” vs. “Never” (β = −2.90 ± 1.17, *p* = 0.04)) only among Whites was inversely related to HEI-2010 total score. Total HEI-2010 score was also related to caring (Model 1), for the elderly over “5 years vs. Never” among Whites (−7.31 ± 3.54, *p* = 0.04, Model 2). Longitudinally, we found slight potential improvement in diet quality over time (“Daily or Weekly” vs. Never by TIME interaction: +0.88 ± 0.38, *p* = 0.02) with frequent caregiving among Whites, but not so among AAs. Conclusions: Frequent caring for grandchildren had an inverse relationship with the diet quality of White and AA urban middle-aged caregivers, while caring for elderly was inversely linked to diet quality among Whites only. Longitudinal studies should address the paucity of research on caregivers’ nutritional quality.

## 1. Introduction

A study from the National Alliance for Caregiving and American Association of Retired Persons (AARP) concluded that the number of unpaid family caregivers increased by 9.5 million from 2015 to 2020. This increase brings the total number of caregivers in the United States to 53 million. This report, Caregiving in the U.S. 2020, also revealed that family caregivers are facing growing social isolation [1]. Unpaid caregivers provide a range of services from food shopping and preparation to housekeeping and transportation [2,3,4,5,6,7]. According to Healthy People 2020, caregivers are at increased risk for adverse health consequences [2].

Numerous studies suggest a sizeable burden ascribed to caregiving for older persons [3,4,5,6,7,8,9]. In fact, the poor nutritional status of an older care recipient is independently more associated with caregiver burden than cognitive and physical disability [3]. Providing care to older community-dwelling adults may also adversely impact the caregiver’s diet quality. There is growing empirical evidence that links such behaviors as healthful eating, regular physical activity, and stress reduction to well-being [10,11,12,13,14,15]. The absence of these recommended health behaviors combined with frequent caregiving responsibilities could lead to obesity, hypertension, diabetes, cardiovascular disease, and an array of chronic diseases among caregivers.

Despite some research focused on caregivers with the diet quality of children [16] and nutritional status of the elderly population receiving care [17], little is known about the dietary behavior and quality of the caregivers themselves in non-clinical settings [3]. Most of the studies explored cross-sectional associations and there is a significant lack of longitudinal research in the caregiving literature. The primary aim of this study is to advance research on caregivers’ health behaviors by analyzing caregivers’ frequencies of caregiving in relation to their diet quality. We considered care receivers of all age groups and relationship to the caregiver, including “children or grandchildren” and “elderly persons,” thus exploring differential risk of poor dietary behavior depending on the type of caregiving. A second aim was to further examine caregiving × diet quality components, separately among Whites and African Americans. Based on the existing literature of disproportionate caregiving burden in African Americans [18,19,20], we hypothesize that diet quality will be more negatively associated than their White counterparts. To the best of our knowledge, this is the first investigation among community-dwelling middle-aged adults as caregivers in relation to caregiver diet quality.

## 2. Methods

### 2.1. Data Source

The Healthy Aging in Neighborhoods of Diversity Across the Life Span (HANDLS) study [21] investigates health disparities in an area probability sample of socioeconomically diverse working age African American and White adults in Baltimore, Maryland. The study began in 2004 and the initial cohort consisted of 3720 men and women (30–64 years). Data in waves 3 and 4 were collected during in-person examinations on mobile research vehicles (MRV) parked in the community. Our present study uses dietary recalls from waves 3 (2009–2013) and 4 (2013–2017), and caregiver data collected during the examinations.

### 2.2. Participants

*Cross-sectional sample.* Up to 2019 participants had complete caregiver data at wave 4 of whom 1945 had complete wave 4 dietary data (w4 sample).

*Longitudinal sample.* Up to 1696 participants had complete caregiver data at wave 3 of whom 1674 had dietary data at either waves 3 or 4. Of those 1624 had dietary data at wave 3 (w3 sample), and 1359 had dietary data at wave 4. W3 and w4 samples were used for descriptive purposes. The longitudinal analysis included 906 and 1674 participants, for questions 6 and 3, respectively, with up to two repeats on the Healthy Eating Index 2010 (HEI-2010, mean observations/participant, k = 1.8).

We examined whether exclusions for missing dietary data represented the sample with complete caretaking data in wave 4. There were no significant differences in the distributions of age (*p* = 0.37), sex (*p* = 0.24), educational attainment (*p* = 0.06), or body mass index (BMI, *p* = 0.62) associated with excluding 74 participants without dietary data.

### 2.3. Dietary Methods and Quality

We collected 24 h dietary recalls using the United States Department of Agriculture (USDA) computerized automated multiple-pass method (AMPM) [22]. The AMPM was designed with cues and prompts to help the participant recall all beverages and foods in the past 24 h and is described elsewhere in detail (21). Trained dietary interviewers conducted all the dietary recalls which were scheduled approximately 4–10 days apart. The first recall was collected in person at MRV parked in the community and the second, by telephone. Participants used an illustrated food Model booklet to estimate accurate quantities of foods and beverages consumed for both recalls. Each recall was coded using the USDA Survey Net data processing system to match the foods with codes in the Food and Nutrient Database for Dietary Studies [23].

### 2.4. Primary Outcome: Healthy Eating Index 2010 (HEI-2010)

Food-based diet quality was assessed by HEI-2010 scores, calculated from 24 h recalls. The National Cancer Institute’s Applied Research website provided the basic steps for calculating the HEI-2010 component and total scores and statistical codes for 24 h dietary recalls [24]. A detailed description of the procedure used for this study is available on the HANDLS website [25]. Component and total HEI-2010 scores were calculated for each recall day and were averaged to obtain a mean value for each wave.

### 2.5. Primary Exposure: Caregiver Status

Caregiver questions were administered to HANDLS participants as part of an audio computer-assisted self-interviewing questionnaire. For the present study, three items examined caregiving as “caring for grandchildren,” “unpaid care for others,” and, “duration of elder care.” The questions related to caregiving for grandchildren and others were identical in waves 3 and 4. After participants acknowledged they had grandchildren they were asked “How often do you spend time caring for your grandchildren?” (defined in this article as “caring for grandchildren”). The possible responses were almost every day, once or twice a week, once or twice a month, once or twice a year, or never. Participants were also asked the number of grandchildren for whom they provided care. After participants acknowledging caring for persons other than their biological children and grandchildren, they were asked “How often do you care for people other than your children or grandchildren without pay?” (defined in this article as “unpaid care for others”). The responses were identical to the question about caregiving for grandchildren.

The question “Excluding your biological children and grandchildren (if you have any), do you provide regular care for an elder in your home?” was only asked in wave 4. If a participant responded yes, then the length of time caring for their elder was asked. The possible responses were less than a year, about 1 year, about 2 years, about 3 years, about 4 years, about 5 years, more than 5 years (defined as “duration of elder care”).

The initial response categories yielded sample sizes that were too small to elicit statistically meaningful differences. In our final analyses, we then re-categorized that data based on the frequency of caregiving (Appendix A) for questions on caring for grandchildren and unpaid care for others (questions 3 and 6). In the recoded variable, “Daily” and “Weekly” were combined to represent more frequent caregiving compared to “Monthly” and “Yearly”, indicating less frequent caring compared to “Never” caregivers. We also recoded the questions on caregiving for elders by combining affirmative responses with the number of years (“>5 years”, “1–5 years”, and “<1 years”) in caregiving using “Never” as a reference category for participants who answered “No”.

### 2.6. Covariates

Socio-demographic characteristics included age, sex, race, and poverty status. Initial age at wave 3 was measured in years and included in models as a continuous variable. Race was dichotomized by self-identification as African American or White. Poverty status was dichotomized using the US Census Bureau, below or above 125% of the poverty thresholds for 2004 [26] based on household size and income. Sex, race, and poverty status were fixed covariates obtained from wave 1 cohort data. From our exploratory analyses, we found depression was a mediator rather than a confounder in the models. Therefore, it was not included in the final analyses. Our sample was also missing more than 10% data on employment status and was not eligible for a meaningful imputation. Therefore, we were unable to identify any potential associations.

### 2.7. Statistical Analyses

Analyses were performed by R Version 4.0.1 [27] in several steps. First, we described selected sample characteristics by race. Means of continuous measures were compared using independent sample Student’s *t*-test, while categorical covariate proportions were compared by race using a χ^2^ test of independence. All variables were assessed for outliers and assumptions of normality. To examine cross-sectional relationships between caregiving exposure and the HEI-2010 outcome, concurrently at wave 4, ordinary least square (OLS) linear regression analyses were conducted. In addition, to assess the association between wave 3 caregiving exposure and wave 3 HEI-2010 outcome, as well as the relationship between wave 3 caregiving exposures and annual rate of change in HEI-2010 between waves 3 and 4, a series of mixed-effects linear regression analyses were conducted. In each model, and for each stratum, the main outcome was HEI-2010 total score with up to two repeats at waves 3 and/or 4 and predictors were each of 2 categorical exposures (questions 3: “Caring for grandchildren” and 6: “Unpaid care for others”) measured at wave 3. All models incorporated number of years elapsed between visits (TIME) and 2-way interaction terms between exposure or covariates with TIME. The model adjusted wave 3 HEI-2010 as well as annualized change over time in this outcome for potentially confounding covariates. Modeling was conducted in a stepwise manner for linear regression models, whereby Model 1 did not adjust for any covariates, while model 2 adjusted for age, sex and poverty status. For mixed-effects linear regression models, only Model 2 was presented. All models were stratified by race due to existing evidence of racial disparity on nutritional status. Furthermore, to test associations between wave 4 caregiver status and wave 4 diet quality measures (HEI-2010 total score), we conducted similar analysis for each caregiver question, adjusted for the same covariates and stratified by race. Parameter estimates from regression models and test statistics are expressed as (β ± Standard Errors, *p*-value), and type I error rate was set at 0.05 for most analyses, with *p* < 0.10 considered to be a trend.

As a secondary analysis, we also examined components of HEI-2010 as an outcome in relation to the caring for grandchildren question (i.e., question 3) as the exposure, while stratifying by race and adjusting for multiple covariates, namely age, sex, and poverty status. The same approach was applied to the unpaid care for others question (i.e., question 6) as the main exposure. This analysis consisted of series of multiple mixed-effects linear regression models with HEI-2010 component outcome measured at waves 3 and/or 4, while caregiver status exposure was measured at wave 3. Given the multiplicity of tests for HEI-2010 components, *p*-values associated with key parameters (main effects of exposures at wave 3 on initial and change in outcomes between waves 3 and 4) were adjusted using familywise Bonferroni correction. This resulted in an adjusted *p*-value of 0.05/12 = 0.004 for HEI-2010’s component analysis, as opposed to 0.05 for previous analyses.

To visualize key findings from mixed-effects linear regression models, predictive margins of the HEI-2010 total score was plotted across TIME for select exposures and racial strata for which findings were deemed statistically significant. In the figure, trajectories were compared with the common referent “Never” in terms of initial predicted HEI-2010 mean and its predicted slope over time for each level of exposure, accounting for covariates included in the model.

## 3. Results

### 3.1. Sample Characteristics

Individuals who had complete HEI-2010 data in Wave 3 numbered 1624 with 58% African Americans (Table 1). Mean age was 52.8 years (~58% women) with no differences by race detected in terms of age (*p* = 0.17) and sex (*p* = 0.93), employment status (*p* = 0.10) or depression (0.08). Among both Whites and African Americans, the sample consisted of individuals living above 125% poverty (~70% of Whites, and 57.0% of African Americans) with statistically significant differences (*p* < 0.001) in comparison to those with income lower than 125% poverty status. HEI-2010 total score did not vary by race (*p* = 0.64) and had a mean of 46.0 out of a maximum of 100. Racial differences were detected at waves 3 and 4, with respect to the question related to “caring for grandchildren” (*p* < 0.001) while the question on “unpaid care for others” (*p* = 0.63 and *p* = 0.64 respectively) showed no difference between Whites and African Americans (Table 1 and Table 2). The question related to duration of caregiving for the elders was also independent of race (*p* = 0.63) at wave 4 (N = 1945) (Table 2).

### 3.2. HEI-2010 and Caregiver Status at Wave 4

The cross-sectional analysis using linear regression models was performed only at wave 4, for participants with complete dietary and select caregiver status measures at wave 4. Among Whites, frequent caregiving (“Daily or Weekly” vs. “Never”) for grandchildren was associated with a lower total score on HEI-2010 (poorer diet quality) both in Model 1 (β ± SE, *p*: −2.90 ± 1.17, *p* = 0.02) and fully adjusted Model 2 (β = −2.83 ± 1.19, *p* = 0.03). Similarly, among African Americans, frequent caring for grandchildren (“Daily or Weekly” vs. “Never”) was inversely related to HEI-2010 total score, reflecting poor diet quality in Model 2 (β = −1.84 ± 0.79, *p* = 0.02) (Table 3).

There were no significant associations between unpaid care for others and diet quality for either race (Table 3). Among Whites, “duration of elder care” was inversely associated with HEI-2010 total score (β = −7.31 ± 3.54, *p* = 0.02), when caring for more than 5 years, but only in the unadjusted model (Model 1, Table 3).

### 3.3. HEI-2010 (Waves 3 and 4) and Longitudinal Caregiver Status

**Figure 1:**
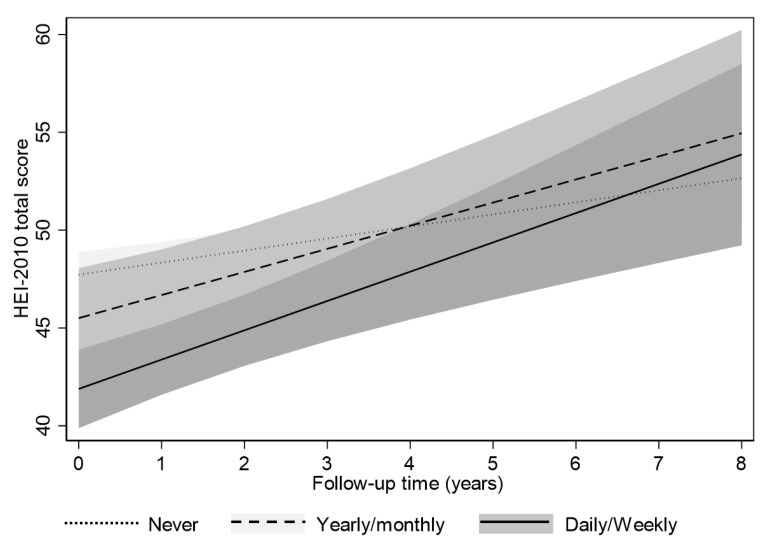
Predictive margins with 95% confidence intervals (CI) of HEI-2010 total score over time, across frequency of caregiving for grandchildren (question 3) among Whites. *Note: predictive margins were created with 95%CI using fixed portion of the mixed effects regression model, adjusting for age, sex and poverty status, Model 2 (*Table 4*).*

Results from mixed-effects linear regression models (Table 4), with wave 3 “caring for grandchildren” as exposure demonstrated significant findings, only in Whites. In fact, “Daily or Weekly” vs. “Never” caring for grandchildren at wave 3 was inversely associated with wave 3 HEI-2010 total score (γ ± SE, *p*: −5.83 ± 1.21, *p* < 0.0001) but positively associated with longitudinal change in this diet quality score between waves 3 and 4 (+ 0.88 ± 0.38, *p* = 0.02) in Whites. The latter finding is at odds with our hypothesis. The exposure-outcome relationships from this mixed-effects linear regression model are illustrated using predictive margins (Figure 1), showing the trajectory of HEI-2010 over time across the “caring for grandchildren” exposure levels, and specifically among Whites. The figure clearly shows the reduced diet quality with more frequent caring for grandchildren at initial visit, coupled with improvement of diet quality over time at the “Daily or Weekly” frequency of caregiving in Whites. No such association patterns were detected among African Americans. There were also no associations detected, neither cross-sectionally nor longitudinally, with respect to the “unpaid care for others” exposure, and for either racial group.

### 3.4. Caregiver Status and Association with HEI-2010 Components

According to findings from Supplementary Table S1, higher frequency of care for grandchildren (“Daily or Weekly” vs. “Never”) was associated with better diet quality among Whites for sodium (β = +0.73 ± 0.28, *p* = 0.009) but only in the initial wave (wave 3). Other cross-sectional associations revealed poor diet quality for the HEI-2010 components of total vegetables (β = −0.29 ± 0.13, *p* = 0.03) greens and beans (β = −0.33 ± 0.15, *p* = 0.04), total fruits (β = −0.43 ± 0.16, *p* = 0.006), whole fruits (β = −0.56 ± 0.17, *p* = 0.04), whole grains (β = −0.89 ± 0.24, *p* = 0.002), total dairy (β = −0.56 ± 0.25, *p* = 0.03), seafood and plant protein (β = −0.53 ± 0.16, *p* = 0.03), and solid fat and added sugar calories (−2.01 ± 0.53, *p* = 0.000). Wholes grains (β = −0.67 ± 0.29, *p* = 0.002) and seafood and plant protein (β = −0.34 ± 0.20, *p* = 0.08) was also inversely related to “Monthly or Yearly” vs. “Never” caring for grandchildren, also among Whites.

In contrast, “Daily or Weekly” vs. “Never” caregiving for grandchildren in Whites showed a slight longitudinal increase over time in total protein (β = +0.11 ± 0.04, *p* = 0.004), fatty acid (β = +0.34 ± 0.11, *p* = 0.001) and solid fat and added sugar (β = +0.38 ± 0.18, *p* = 0.03) intake. The seafood and plant protein component (β = +0.15 ± 0.07, *p* = 0.04) was higher among Whites in caring for grandchildren “Monthly or Yearly” vs. “Never”.

Among African American caregivers in the “Daily or Weekly” vs. “Never” group for caring for grandchildren total fruits (β = −0.22 ± 0.13, *p* = 0.09) and in “Monthly or Yearly” vs. “Never” group, whole fruits (β = −0.29 ± 0.13, *p* = 0.03) and sodium (β = −0.43 ± 0.23, *p* = 0.06) associations were of note. The whole fruits (β = +0.08 ± 0.04, *p* = 0.02) and total dairy (β = +0.19 ± 0.06, *p* = 0.001) components were the only two showing a faster rate of increase over time among African American caregivers in the “Monthly or Yearly” vs. “Never” group for caring for grandchildren.

Unpaid care for others (Supplementary Table S2) demonstrated one inverse longitudinal association with refined grains in African Americans (β = −0.42 ± 0.16, *p* = 0.008) for the “Monthly or Yearly” vs. “Never” caregiving exposure.

## 4. Discussion

Providing care for grandchildren almost every day or once or twice a week was inversely associated with diet quality among Whites and African Americans, cross-sectionally. Additionally, but only in Whites, was caring for grandchildren “Daily or Weekly” associated with greater improvement in diet quality, as evidenced by increases in HEI-2010 scores for the total protein, fatty acid, and solid fat and added sugar calorie components over time. Less frequent care of grandchildren was associated with potentially greater improvement in HEI-2010 scores for seafood and plant proteins among White caregivers vs. AA caregivers. Similar results were observed for whole fruit and the dairy consumption among African American caregivers compared to White caregivers. In addition, caring for the elderly, over 5 years was negatively associated with diet quality but only among Whites. Caregivers for the elderly in our population spent anywhere between less than 1 year to 5 years and beyond in their service. We found that maximum duration of caring for the elderly had the most negative impact on diet quality. To the best of our knowledge, this is the first report on community-dwelling adults as caregivers and their diet quality.

Caregiver burden has been a poorly defined concept to date, mostly focusing on caregivers of clinical populations with Alzheimer’s Disease, Multiple Sclerosis, cancer, and other terminal illnesses. Caregiver burden was defined in an article about families with a patient with multiple sclerosis as “… a multidimensional response to physical, psychological, emotional, social, and financial stressors associated with the caregiving experience” [28]. It is possible that the burden imposed by caregiving can lead to higher risk of depression and a lower quality of life including poor quality diet and exercise. While nutrition has been emphasized at an organizational level, such as by the American Cancer Society and AARP, we were unable to find health guidelines for caregivers that focus on nutritional status specifically. Most of the programs and current research do not mention caregiver nutrition as part of the overall caregiver burden [29]. This research, therefore, sheds light on a crucial area essential to optimal caregiving.

Consistent with the report by Cohen and colleagues [30], there were more African American and White women compared to African American and White men providing care in the HANDLS study. Our findings support the original hypothesis that caregiving will be associated with poor diet quality within African American. However, the study findings did not support our hypothesis that the diet quality of Whites would be better than African Americans. Although there is a lack of published diet-related data for caregivers, there is evidence for sociocultural factors influences on racial differences in caregiving [31,32,33,34,35]. For instance, African American caregivers have reported more traditional ideology, namely caregiving as a responsibility, compared to White caregivers [32]. In addition, African Americans with a single caregiving role had better self-rated health compared to non-caregivers, while other racial groups with multiple caregiving roles had better self-rated health than non-caregivers [33].

The influence of grandparents on dietary intake of their grandchildren has been documented by others [36,37,38,39,40]. Jongenelis and colleagues reported that grandparents are important stakeholders in the promotion of favorable dietary behaviors [40].The impact depends to a large extent on the nutrition knowledge, attitudes and beliefs of their caregivers [41]. Based on a research with low-income African American families in Baltimore, Wingert and colleagues suggested children who were present during grocery shopping have the potential to increase the purchase of healthful items by caregivers [42], although shopping with children promoted unplanned, unhealthy food choices. This opposite association might partially explain the inverse association of time spent in caring for grandchildren and HEI-2010 scores we observed. In our sample, caregiving of children adversely impacted diet quality.

In an additional longitudinal HEI-2010 component analysis, significant findings were found for 5 components among White caregivers and 2 components among African American caregivers. Caring for grandchildren “Daily or Weekly” vs. “Never” was associated with improved diet quality for total protein, fatty acid, and solid fat and added sugar intakes among only Whites. Other studies have predicted similar changes in consumption of empty calories with caring for grandchildren [36,43]. Additional improvements were seen in seafood and plant protein intake over time, also among Whites caring for grandchildren “Monthly or Yearly” vs. “Never”. The HEI-2010 component score for whole fruits and total dairy intakes improved over time in African American caregivers, while caring for grandchildren either “Monthly or Yearly” vs. “Never”.

The slight improvement in whole fruits intake over time among African Americans might be partially explained by the findings of a report by Li and colleagues. They found substantially different food consumption and purchasing behaviors between women urban-dwelling African Americans and Whites in the US [20]. The racial differences between the older African American and White women were found attributable to socioeconomic and behavioral differences rather than geographical variations. That is because the study population came from a densely populated urban area like HANDLS. In addition, the African American older women were particularly interested in obtaining healthier foods e.g., lean meat and low sodium products for health reasons [20]. Our sample being like the subjects studied, we suspect similar mindset could have influenced the improvement in whole fruits intake in HANDLS. The improvement in dairy consumption among African American caregivers over time is positive since previous studies report inadequate consumption of dairy in this group [44,45].

Our interest in caregiver nutritional status lies in the fact that poor diet quality is associated with numerous adverse health outcomes among older adults. Previously in HANDLS, we have demonstrated that poor diet quality is associated with poorer cognitive outcome both in the short and long run [46,47] with a risk of malnutrition over time [48]. Preventing or delaying the onset of mild cognitive impairment (MCI) can lead to a substantial improvement in quality of life. In fact, studies linking behavioral factors with cognitive performance demonstrated that a diverse diet with good supply of macro- and micro-nutrients [49], reduced alcohol intake [50] and increased physical activity [51,52] are helpful in attenuating MCI [53] and progression to AD in older adults [54,55]. More specifically, growing evidence supports the protective role of diets rich in fish, heart-healthy oils, fresh fruits and vegetables in reducing risk for MCI [56,57,58] as well as early stages of dementia [59]. Healthier food choices among caregivers could lower the demonstrated caregiver burden on family or other unpaid caregivers [5].

Our study has several strengths. The HANDLS study has a diverse sample of both White and African American adults who are often unavailable for side-by-side comparisons in large cohorts, particularly observational longitudinal studies. Diet quality was based on two 24 h recalls in contrast with other studies which rely on only one recall often with a far less comprehensive survey instrument. This is also the first study to look at diet quality, both total and components of HEI of caregivers in a socially diverse, community cohort. Additionally, our study had a large sample size to analyze various tested associations and for detected associations to survive multiple testing. Lastly, we presented both cross-sectional and longitudinal analyses to support our hypothesis. Therefore, the present study makes a unique contribution to the nutrition and caregiving literature, simultaneously.

Our study is not without limitations. Due to the observational nature of the analysis, we cannot infer causality. Another limitation is our reliance on participants’ 24 h dietary recall. We were unable to obtain information on cooking or meal preparation habits and perceived diet perception in this study, which could have influenced the decision-making of the caregivers. Despite adjusting for multiple testing, the role of chance cannot be ruled out.

Our findings need further support from additional research, especially around perceptions of healthy eating and cooking behavior. Future studies are necessary to test such associations in a larger, more heterogeneous samples with respect to diet quality and care. There is also a need to consider factors that promote low diet quality among higher-SES groups in assessing similar associations.

## 5. Conclusions

Overall, our findings give valuable insights into the associations between caregiving frequency and diet quality in urban adults and how the association varies by race. Although diet quality, evaluated by HEI-2010, reflected only the achievement of about 50% of recommendations, caring for grandchildren was associated with a faster rate of increase in diet quality over time, while caring for elders for more than 5 years was linked to faster decrements in diet quality over time in White urban caregivers.

## Figures and Tables

**Table 1:** Characteristics of Healthy Aging in Neighborhoods of Diversity Across the Life Span (HANDLS) participants at wave 3 (N = 1624).

	Whites	African Americans	*p* ^1race^
	**(N = 675)**	**(N = 949)**	
Age at wave 3 (year), Mean (SD)	52.72 (8.83)	52.47 (8.84)	0.17
Sex, N (%)			0.89
Women	395 (58.5)	552 (58.2)	
Men	280 (41.5)	397 (41.8)	
Poverty Status, N (%)			<0.001
Above 125%	469 (69.5)	541 (57.0)	
Below 125%	206 (30.5)	408 (43.0)	
	**(N = 664)**	**(N = 852)**	
Employment Status, N (%)			0.094
Yes	346 (52.1)	407 (47.8)	
No	318 (47.9)	445 (52.2)	
	**(N = 669)**	**(N = 943)**	
Depression, N (%)			0.069
Yes	292 (43.6)	369 (39.1)	
No	377 (56.4)	574 (60.9)	
	**(N = 675)**	**(N = 949)**	
Healthy Eating Index (HEI)-2010 score	46.14 (13.04)	45.87 (10.98)	0.64
**Caregiver Data**			
Caring for grandchildren, N (%)	**(N = 675)**	**(N = 949)**	<0.001
Daily vs. Weekly	148 (21.9)	273 (28.8)	
Monthly vs. Yearly	91 (13.5)	194 (20.4)	
Never	436 (64.6)	482 (50.8)	
Unpaid care for others, N (%)	**(N = 303)**	**(N = 578)**	0.63
Daily vs. Weekly	37 (12.2)	71 (12.3)	
Monthly vs. Yearly	10 (3.3)	27 (4.7)	
Never	256 (84.5)	480 (82.9)	

^1race^ *p*-value associated with null hypothesis of no difference by race based on *t*-test for continuous variables and chi-square test for categorical variables.

**Table 2:** Characteristics of HANDLS participants at wave 4 (N = 1945).

	Whites	African Americans	*p* ^1race^
	**(N = 771)**	**(N = 1174)**	
Age at wave 4 (year), Mean (SD)	56.58 (9.03)	56.56 (9.15)	0.10
Sex, N (%)			0.84
Women	456 (59.1)	689 (58.7)	
Men	315 (40.9)	485 (41.3)	
Poverty Status, N (%)			<0.001
Above 125%	518 (67.2)	652 (55.5)	
Below 125%	253 (32.8)	522 (44.5)	
	**(N = 724)**	**(N = 1106)**	
Employment Status, N (%)			0.83
Yes	315 (43.5)	487 (44.0)	
No	409 (56.5)	619 (56.0)	
	**(N = 739)**	**(N = 1135)**	
Depression, N (%)			0.064
Yes	289 (39.1)	396 (34.9)	
No	450 (60.9)	739 (65.1)	
	**(N = 771)**	**(N = 1174)**	
HEI-2010 score	48.97 (13.1)	48.58 (11.4)	0.49
**Caregiver Data**			
Caring for grandchildren, N (%)	**(N = 750)**	**(N = 1156)**	<0.001
Daily vs. Weekly	173 (23.1)	361 (31.2)	
Monthly vs. Yearly	117 (15.6)	283 (24.5)	
Never	460 (61.3)	512 (44.3)	
Unpaid care for others, N (%)	**(N = 735)**	**(N = 1138)**	0.64
Daily vs. Weekly	82 (11.2)	115 (10.1)	
Monthly vs. Yearly	26 (3.5)	47 (4.1)	
Never	627 (85.3)	976 (85.8)	
Duration for elder care, N (%)	**(N = 763)**	**(N = 1169)**	0.63
>5 year	14 (1.8)	28 (2.4)	
1–5 year	29 (3.8)	40 (3.4)	
<1 year	9 (1.2)	20 (1.7)	
Never	711 (93.2)	1081 (92.5)	

^1race^ *p*-value associated with null hypothesis of no difference by race based on *t*-test for continuous variables and chi-square test for categorical variables.

**Table 3:** Cross-sectional associations (Wave 4 only) between caregiving measures (recoded questions 3, 6 and 7/8) and diet quality measured with total HEI-2010 score (β ± SE, *p*-value), stratified by race, for HANDLS participants: ordinary least square (OLS) linear regression ^1^.

	Whites	African Americans
***Y = HEI-2010 Total Score***	**(N = 750)**	**(N = 1156)**
*X = Caring for grandchildren*		
Model 1		
Daily or Weekly	−2.90 ± 1.17 **	−0.86 ± 0.78
Monthly or Yearly	+0.60 ± 1.35	+0.53 ± 0.84
Model 2		
Daily or Weekly	−2.83 ± 1.19 **	−1.84 ± 0.79 **
Monthly or Yearly	+0.18 ± 1.36	−0.86 ± 0.85
***Y = HEI-2010 total score***	**(N = 735)**	**(N = 1138)**
*X = Unpaid care for others*		
Model 1		
Daily or Weekly	−2.37 ± 1.54	−1.71 ± 1.12
Monthly or Yearly	−0.36 ± 2.63	−0.80 ± 1.69
Model 2		
Daily or Weekly	−2.00 ± 1.52	−1.02 ± 1.10
Monthly or Yearly	+0.29 ± 2.57	+0.24 ± 1.66
***Y = HEI-2010 total score***	**(N = 763)**	**(N = 1169)**
*X = Duration for elder care*		
Model 1		
<1 year	−4.05 ± 4.40	−1.84 ± 2.57
1–5 year	−1.84 ± 2.48	+2.07 ± 1.83
>5 year	−7.31 ± 3.54 **	−0.24 ± 2.18
Model 2		
<1 year	−2.58 ± 4.32	−1.27 ± 2.52
1–5 year	−1.39 ± 2.43	+1.88 ± 1.80
>5 year	−6.45 ± 3.49	−0.10 ± 2.13

** *p* < 0.05, for null hypothesis that OLS regression coefficient β = 0. ^1^ Models were stratified by race. Model 1: unadjusted; Model 2: were adjusted for age at wave 4, sex, and poverty status. The main exposure variables HEI-2010, was from wave-4 only, for the cross- sectional analyses. Models included recoded categorical questions 3 or 6 or 7/8 as main exposure variables (X), alternatively. N = number of participants, X = main exposure, Y = outcome variable.

**Table 4:** Longitudinal Associations of caregiving exposures (X = recoded questions 3 and 6) with diet quality (Y = HEI-2010 total score) over time, stratified by race, for HANDLS participants (N = 1674) [γ ± SE, *p*-value]: Mixed-effects linear regression models ^1^.

***Y = HEI-2010 Total Score***	**Whites (N = 697, k = 1.8)**	**African Americans (N = 977, k = 1.8)**
Time	+0.73 ± 0.23 ***	+0.56 ± 0.19 ***
X = Caring for grandchildren		
Daily or Weekly	−5.83 ± 1.21 ***	−1.13 ± 0.83
Monthly or Yearly	−2.22 ± 1.45	−0.31 ± 0.92
Caring for grandchildren (X) × Time		
Daily or Weekly	+0.88 ± 0.38 *	+0.04 ± 0.23
Monthly or Yearly	+0.56 ± 0.42	+0.11 ± 0.25
***Y = HEI-2010 Total Score***	**Whites (N = 311, k = 1.8)**	**African Americans (N = 595, k = 1.8)**
Time	+1.99 ± 0.42 ***	+0.61 ± 0.23 ***
X = Unpaid care for others		
Daily or Weekly	−1.39 ± 1.94	−0.53 ± 1.35
Monthly or Yearly	−1.42 ± 3.50	+1.71 ± 2.10
Unpaid care for others (X) × Time		
Daily or Weekly	−0.09 ± 0.74	−0.04 ± 0.35
Monthly or Yearly	+0.28 ± 1.41	−1.11 ± 0.59

*** *p* < 0.01, * *p* < 0.10 for null hypothesis that fixed effect γ = 0. ^1^ Models were stratified by race and adjusted for age at wave 3, sex, and poverty status. The main outcome variable HEI-2010, was from waves 3 and 4. Models included recoded categorical questions 3 or 6 as main exposure variables (X), alternatively. N = number of participants selected in each model, k = mean number of observations/participant, γ = fixed effect, X = main exposure, Y = outcome variable.

## Data Availability

Data are available upon request to researchers with valid proposals who agree to the confidentiality agreement as required by our Institutional Review Board. We publicize our policies on our website https://handls.nih.gov (accessed on 25 May 2021). Requests for data access may be sent to the PIs or the study manager, Jennifer Norbeck at norbeckje@mail.nih.gov. These data are owned by the National Institute on Aging at the National Institutes of Health. The Principal Investigators have restricted public access to these data because: (1) the study collects medical, psychological, cognitive, and psychosocial information on racial and poverty differences that could be misconstrued or willfully manipulated to promote racial discrimination; and (2) although the sample is fairly large, there are sufficient identifiers that the PIs cannot guarantee absolute confidentiality for every participant as we have stated in acquiring our confidentiality certificate.

## Supplementary Materials

The following are available online at https://www.mdpi.com/article/10.3390/nu13061803/s1.

## Appendix A. Selected Caregiver Questions by Wave in HANDLS

**Care Recipient**	**Questions**	**Response Categories**	**Recoded Categories**	**Referent Category**	**Wave**
Caring for grandchildren	Time spent caring for grandchildren	Daily|Weekly|Monthly|Yearly|Never	Daily or Weekly; Monthly or Yearly; Never	Never	3 and 4
Unpaid care for others	How often provide unpaid care for others, not children and grandchildren	Daily|Weekly|Monthly|Yearly|Never	Daily or Weekly; Monthly or Yearly; Never	Never	3 and 4
Duration for elder care	Excluding your biological children and grandchildren (if you have any), do you provide regular care for an elder in your home?	No|Yes	N/A	N/A	4 only
	How long have you been caring for your elder?	<1 year|1 year|2 years|3 years|4 years|5 years|>5 years	<1 year; 1–5 years: >5 years	Never	4 only

## References

[1] (2021). The National Alliance for Caregiving Research (NACR). https://www.caregiving.org/research/.

[2] (2020). Promotion OoDPaH: Healthy People 2020. https://www.healthypeople.gov/2020/.

[3] Tana C., Lauretani F., Ticinesi A., Gionti L., Nouvenne A., Prati B., Meschi T., Maggio M. (2019). Impact of Nutritional Status on Caregiver Burden of Elderly Outpatients. A Cross-Sectional Study. Nutrients.

[4] Anderson L.A., Edwards V.J., Pearson W.S., Talley R.C., McGuire L.C., Andresen E.M. (2013). Adult caregivers in the United States: Characteristics and differences in well-being, by caregiver age and caregiving status. Prev. Chronic Dis..

[5] Catherine Riffin P.H.V.N., Jennifer L.W., Terri F. (2017). Family and Other Unpaid Caregivers and Older Adults with and without Dementia and Disability. J. Am. Geriatr. Soc..

[6] Ysseldyk R., Kuran N., Powell S., Villeneuve P.J. (2019). Self-reported health impacts of caregiving by age and income among participants of the Canadian 2012 General Social Survey. Health Promot. Chronic Dis. Prev. Can. Res. Policy Pract..

[7] Ross A., Sundaramurthi T., Bevans M. (2013). A Labor of Love: The Influence of Cancer Caregiving on Health Behaviors. Cancer Nurs..

[8] Rha S.Y., Park Y., Song S.K., Lee C.E., Lee J. (2015). Caregiving burden and health-promoting behaviors among the family caregivers of cancer patients. Eur. J. Oncol. Nurs..

[9] Bevans M., Sternberg E.M. (2012). Caregiving burden, stress, and health effects among family caregivers of adult cancer patients. JAMA.

[10] Navarro F. (2011). Diet, Exercise, Mindfulness, and Relaxation: Stress Management and Stress Reduction.

[11] Martinez-Gonzalez M.A., Gea A., Ruiz-Canela M. (2019). The Mediterranean Diet and Cardiovascular Health. Circ. Res..

[12] Warburton D.E.R., Bredin S.S.D. (2019). Health Benefits of Physical Activity: A Strengths-Based Approach. J. Clin. Med..

[13] Sharma M., Rush S.E. (2014). Mindfulness-based stress reduction as a stress management intervention for healthy individuals: A systematic review. J. Evid. Based Complement. Altern. Med..

[14] Ritchie C.S., Burgio K.L., Locher J.L., Cornwell A., Thomas D., Hardin M., Redden D. (1997). Nutritional status of urban homebound older adults. Am. J. Clin. Nutr..

[15] Tucker C.M., Butler A.M., Loyuk I.S., Desmond F.F., Surrency S.L. (2009). Predictors of a health-promoting lifestyle and behaviors among low-income African American mothers and white mothers of chronically ill children. J. Natl. Med. Assoc..

[16] Morgan E.H., Schoonees A., Sriram U., Faure M., Seguin-Fowler R.A. (2020). Caregiver involvement in interventions for improving children’s dietary intake and physical activity behaviors. Cochrane Database Syst. Rev..

[17] Correa B., Leandro Merhi V.A., Pagotto Fogaca K., de Oliveira M.R.M. (2009). Caregiver’s education level, not income, as determining factor of dietary intake and nutritional status of individuals cared for at home. J. Nutr. Health Aging.

[18] Henry J.L., Trude A.C.B., Surkan P.J., Anderson Steeves E., Hopkins L.C., Gittelsohn J. (2018). Psychosocial Determinants of Food Acquisition and Preparation in Low-Income, Urban African American Households. Health Educ. Behav..

[19] Beydoun M.A., Wang Y. (2008). How do socio-economic status, perceived economic barriers and nutritional benefits affect quality of dietary intake among US adults?. Eur. J. Clin. Nutr..

[20] Li W., Youssef G., Procter-Gray E., Olendzki B., Cornish T., Hayes R., Churchill L., Kane K., Brown K., Magee M.F. (2017). Racial Differences in Eating Patterns and Food Purchasing Behaviors among Urban Older Women. J. Nutr. Health Aging.

[21] Evans M.K., Lepkowski J.M., Powe N.R., LaVeist T., Kuczmarski M.F., Zonderman A.B. (2010). Healthy aging in neighborhoods of diversity across the life span (HANDLS): Overcoming barriers to implementing a longitudinal, epidemiologic, urban study of health, race, and socioeconomic status. Ethn. Dis..

[22] Moshfegh A.J., Rhodes D.G., Baer D.J., Murayi T., Clemens J.C., Rumpler W.V., Paul D.R., Sebastian R.S., Kuczynski K.J., Ingwersen L.A. (2008). The US Department of Agriculture Automated Multiple-Pass Method reduces bias in the collection of energy intakes. Am. J. Clin. Nutr..

[23] FNDDS. https://www.ars.usda.gov/northeast-area/beltsville-md-bhnrc/beltsville-human-nutrition-research-center/food-surveys-research-group/docs/fndds/.

[24] HEI Tools for Researchers. https://epi.grants.cancer.gov/hei/tools.html.

[25] Healthy Eating Index 2010. https://handls.nih.gov/06Coll-w01HEI.htm.

[26] Bureau UC: US Census Bureau (2004). Social, Economic, and Housing Statistics Division. Poverty Thresholds. https://www.census.gov/data/tables/time-series/demo/income-poverty/historical-poverty-thresholds.html.

[27] R Core Team (2019). R: A Language and Environment for Statistical Computing.

[28] Buhse M. (2008). Assessment of Caregiver Burden in Families of Persons with Multiple Sclerosis. J. Neurosci. Nurs..

[29] Tamizi Z., Fallahi-Khoshknab M., Dalvandi A., Mohammadi-Shahboulaghi F., Mohammadi E., Bakhshi E. (2019). Defining the concept of family caregiver burden in patients with schizophrenia: A systematic review protocol. Syst. Rev..

[30] Cohen S.A., Sabik N.J., Cook S.K., Azzoli A.B., Mendez-Luck C.A. (2019). Differences within Differences: Gender Inequalities in Caregiving Intensity Vary by Race and Ethnicity in Informal Caregivers. J. Cross-Cult. Gerontol..

[31] Cook S.K., Cohen S.A. (2018). Sociodemographic Disparities in Adult Child Informal Caregiving Intensity in the United States. J. Gerontol. Nurs..

[32] Sander A.M., Hanks R.A., Ianni P.A., Boileau N.R., Kratz A.L., Hahn E.A., Tulsky D.S., Carlozzi N.E. (2019). Sociocultural Factors Influencing Caregiver Appraisals Following Traumatic Brain Injury. Arch. Phys. Med. Rehabil..

[33] Kim G., Allen R.S., Wang S.Y., Park S., Perkins E.A., Parmelee P. (2019). The Relation Between Multiple Informal Caregiving Roles and Subjective Physical and Mental Health Status Among Older Adults: Do Racial/Ethnic Differences Exist?. Gerontologist.

[34] Rote S.M., Angel J.L., Moon H., Markides K. (2019). Caregiving Across Diverse Populations: New Evidence From the National Study of Caregiving and Hispanic EPESE. Innov. Aging.

[35] Suitor J.J., Gilligan M., Rurka M., Con G., Peng S., Pillemer K. (2018). Conflict with Mothers and Siblings During Caregiving: Differential Costs for Black and White Adult Children. J. Gerontol. Ser. B.

[36] Roberts M., Pettigrew S. (2010). The Influence of Grandparents on Children’s Diets. J. Res. Consum..

[37] Tan B.Q.M., Hee J.M., Yow K.S., Sim X., Asano M., Chong M.F.-F. (2019). Feeding-Related Knowledge, Attitudes, and Practices among Grandparents in Singapore. Nutrients.

[38] Pankhurst M., Mehta K., Matwiejczyk L., Moores C.J., Prichard I., Mortimer S., Bell L. (2019). Treats are a tool of the trade: An exploration of food treats among grandparents who provide informal childcare. Public Health Nutr..

[39] Young H.M., Bell J.F., Whitney R.L., Ridberg R.A., Reed S.C., Vitaliano P.P. (2020). Social Determinants of Health: Underreported Heterogeneity in Systematic Reviews of Caregiver Interventions. Gerontol..

[40] Jongenelis M.I., Morley B., Pratt I.S., Talati Z. (2020). Diet quality in children: A function of grandparents’ feeding practices?. Food Qual. Prefer..

[41] Acheampong I., Haldeman L. (2013). Are nutrition knowledge, attitudes, and beliefs associated with obesity among low-income Hispanic and African American women caretakers?. J. Obes..

[42] Wingert K., Zachary D.A., Fox M., Gittelsohn J., Surkan P.J. (2014). Child as change agent. The potential of children to increase healthy food purchasing. Appetite.

[43] Carthron D.L., Johnson T.M., Hubbart T.D., Strickland C., Nance K. (2010). “Give me some sugar!” The diabetes self-management activities of African-American primary caregiving grandmothers. J. Nurs. Sch..

[44] Fulgoni V., Nicholls J., Reed A., Buckley R., Kafer K., Huth P., DiRienzo D., Miller G.D. (2007). Dairy consumption and related nutrient intake in African-American adults and children in the United States: Continuing survey of food intakes by individuals 1994–1996, 1998, and the National Health And Nutrition Examination Survey 1999–2000. J. Am. Diet. Assoc..

[45] Wang Y., Jahns L., Tussing-Humphreys L., Xie B., Rockett H., Liang H., Johnson L. (2010). Dietary intake patterns of low-income urban african-american adolescents. J. Am. Diet. Assoc..

[46] Hossain S., Beydoun M.A., Kuczmarski M.F., Tajuddin S., Evans M.K., Zonderman A.B. (2019). The Interplay of Diet Quality and Alzheimer’s Disease Genetic Risk Score in Relation to Cognitive Performance Among Urban African Americans. Nutrients.

[47] Hossain S., Beydoun M.A., Weiss J., Kuczmarski M.F., Evans M.K., Zonderman A.B. (2020). Longitudinal associations between dietary quality and Alzheimer’s Disease genetic risk on cognitive performance among African American adults. Br. J. Nutr..

[48] Fanelli Kuczmarski M., Stave Shupe E., Pohlig R.T., Rawal R., Zonderman A.B., Evans M.K. (2019). A Longitudinal Assessment of Diet Quality and Risks Associated with Malnutrition in Socioeconomic and Racially Diverse Adults. Nutrients.

[49] Charlton K.E. (2002). Eating well: Ageing gracefully!. Asia Pac. J. Clin. Nutr..

[50] Daviglus M.L., Plassman B.L., Pirzada A., Bell C.C., Bowen P.E., Burke J.R., Connolly E.S., Dunbar-Jacob J.M., Granieri E.C., McGarry K. (2011). Risk factors and preventive interventions for Alzheimer disease: State of the science. Arch. Neurol..

[51] Moritani T., Akamatsu Y. (2015). Effect of Exericse and Nutrition upon Lifestyle-Related Disease and Cognitive Function. J. Nutr. Sci. Vitam..

[52] Daly R.M., Gianoudis J., Prosser M., Kidgell D., Ellis K.A., O’Connell S., Nowson C.A. (2015). The effects of a protein enriched diet with lean red meat combined with a multi-modal exercise program on muscle and cognitive health and function in older adults: Study protocol for a randomised controlled trial. Trials.

[53] Orsitto G. (2012). Different components of nutritional status in older inpatients with cognitive impairment. J. Nutr. Health Aging.

[54] Wengreen H.J., Neilson C., Munger R., Corcoran C. (2009). Diet quality is associated with better cognitive test performance among aging men and women. J. Nutr..

[55] Flicker L., Lautenschlager N.T., Almeida O.P. (2006). Healthy mental ageing. J. Br. Menopause Soc..

[56] Hardman R.J., Kennedy G., Macpherson H., Scholey A.B., Pipingas A. (2016). Adherence to a Mediterranean-Style Diet and Effects on Cognition in Adults: A Qualitative Evaluation and Systematic Review of Longitudinal and Prospective Trials. Front. Nutr..

[57] Dussaillant C., Echeverria G., Urquiaga I., Velasco N., Rigotti A. (2016). Current evidence on health benefits of the mediterranean diet. Rev. Med. Chile.

[58] Solfrizzi V., Panza F., Frisardi V., Seripa D., Logroscino G., Imbimbo B.P., Pilotto A. (2011). Diet and Alzheimer’s disease risk factors or prevention: The current evidence. Expert Rev. Neurother..

[59] Petersson S.D., Philippou E. (2016). Mediterranean Diet, Cognitive Function, and Dementia: A Systematic Review of the Evidence. Adv. Nutr..

