# Supplementary material for: Caregiver Status and Diet Quality in Community-Dwelling Adults
Source: Nutrients. 2021 May 26;13(6):1803. doi: 10.3390/nu13061803 (PMC8227086; doi:10.3390/nu13061803)
Supplement: Supplementary file 1 [file nutrients-13-01803-s001.zip › Supplementary table 1.docx]

**Supplementary table 1.** Longitudinal Associations of HEI-2010 components with caregiving questions 3, stratified by race, for HANDLS participants (N=1,674) [β±SE, p-value]: Mixed-effects linear regression models^1^

|  | Whites (N=697) | African Americans (N=977) |
| --- | --- | --- |
| **Total Vegetables** |  |  |
| Time | 0.07±0.03 | 0.05±0.03 |
| Caring for grandchildren |  |  |
| Daily or Weekly | **-0.27±0.13**** | 0.08±0.10 |
| Monthly or Yearly | -0.23±0.15 | 0.05±0.11 |
| Caring for grandchildren× Time |  |  |
| Daily or Weekly | 0.02±0.05 | -0.02±0.03 |
| Monthly or Yearly | 0.02±0.06 | -0.01±0.03 |
| **Greens and Beans** |  |  |
| Time | 0.08±0.04 | 0.08±0.03 |
| Caring for grandchildren |  |  |
| Daily or Weekly | **-0.29±0.15**** | 0.08±0.12 |
| Monthly or Yearly | -0.07±0.18 | -0.07±0.13 |
| Caring for grandchildren× Time |  |  |
| Daily or Weekly | -0.07±0.06 | -0.04±0.04 |
| Monthly or Yearly | -0.03±0.07 | -0.02±0.04 |
| **Total Fruits** |  |  |
| Time | 0.07±0.03 | 0.01±0.03 |
| Caring for grandchildren |  |  |
| Daily or Weekly | -0.43±0.16 | -0.22±0.13 |
| Monthly or Yearly | -0.10±0.19 | -0.10±0.14 |
| Caring for grandchildren× Time |  |  |
| Daily or Weekly | -0.06±0.05 | 0.01±0.04 |
| Monthly or Yearly | 0.01±0.06 | 0.01±0.04 |
| **Whole Fruits** |  |  |
| Time | 0.06±0.03 | -0.001±0.03 |
| Caring for grandchildren |  |  |
| Daily or Weekly | **-0.56±0.17**** | -0.22±0.12 |
| Monthly or Yearly | -0.01±0.20 | -0.29±0.13 |
| Caring for grandchildren× Time |  |  |
| Daily or Weekly | -0.06±0.06 | 0.01±0.04 |
| Monthly or Yearly | -0.01±0.06 | **0.08±0.04*** |
| **Whole grains** |  |  |
| Time | -0.08±0.05 | 0.01±0.04 |
| Caring for grandchildren |  |  |
| Daily or Weekly | **-0.89±0.24***** | -0.24±0.18 |
| Monthly or Yearly | **-0.67±0.29**** | 0.03±0.20 |
| Caring for grandchildren× Time |  |  |
| Daily or Weekly | 0.12±0.09 | 0.03±0.05 |
| Monthly or Yearly | 0.05±0.10 | -0.05±0.06 |
| **Total Dairy** |  |  |
| Time | 0.03±0.06 | -0.004±0.05 |
| Caring for grandchildren |  |  |
| Daily or Weekly | **-0.54±0.26*** | 0.05±0.18 |
| Monthly or Yearly | -0.11±0.31 | -0.24±0.20 |
| Caring for grandchildren× Time |  |  |
| Daily or Weekly | -0.06±0.11 | 0.02±0.06 |
| Monthly or Yearly | -0.13±0.12 | **0.19±0.06***** |
| **Total Protein** |  |  |
| Time | -0.04±0.03 | -0.02±0.02 |
| Caring for grandchildren |  |  |
| Daily or Weekly | -0.19±0.10 | 0.08±0.07 |
| Monthly or Yearly | -0.08±0.12 | 0.05±0.07 |
| Caring for grandchildren× Time |  |  |
| Daily or Weekly | **0.12±0.04***** | -0.03±0.02 |
| Monthly or Yearly | 0.07±0.05 | -0.01±0.02 |
| **Seafood and Plant Protein** |  |  |
| Time | 0.008±0.04 | 0.02±0.03 |
| Caring for grandchildren |  |  |
| Daily or Weekly | **-0.53±0.16***** | -0.02±0.13 |
| Monthly or Yearly | -0.34±0.20 | 0.06±0.14 |
| Caring for grandchildren× Time |  |  |
| Daily or Weekly | 0.09±0.07 | -0.03±0.04 |
| Monthly or Yearly | **0.15±0.08*** | -0.03±0.04 |
| **Fatty Acid** |  |  |
| Time | -0.02±0.07 | -0.04±0.05 |
| Caring for grandchildren |  |  |
| Daily or Weekly | -0.41±0.28 | -0.17±0.20 |
| Monthly or Yearly | -0.11±0.33 | 0.21±0.22 |
| Caring for grandchildren× Time |  |  |
| Daily or Weekly | **0.33±0.12***** | 0.01±0.06 |
| Monthly or Yearly | 0.17±0.13 | -0.06±0.07 |
| **Sodium** |  |  |
| Time | 0.10±0.06 | 0.002±0.05 |
| Caring for grandchildren |  |  |
| Daily or Weekly | 0.73±0.28 | -0.29±0.21 |
| Monthly or Yearly | 0.37±0.34 | -0.43±0.23 |
| Caring for grandchildren× Time |  |  |
| Daily or Weekly | -0.17±0.10 | 0.06±0.06 |
| Monthly or Yearly | -0.02±0.11 | 0.009±0.07 |
| **Refined Grain** |  |  |
| Time | 0.14±0.07 | 0.03±0.05 |
| Caring for grandchildren |  |  |
| Daily or Weekly | -0.16±0.27 | -0.16±0.19 |
| Monthly or Yearly | 0.18±0.32 | 0.20±0.21 |
| Caring for grandchildren× Time |  |  |
| Daily or Weekly | 0.11±0.11 | 0.07±0.06 |
| Monthly or Yearly | -0.02±0.12 | -0.03±0.07 |
| **Solid Fat and Added Sugar calories** |  |  |
| Time | 0.50±0.10 | 0.41±0.09 |
| Caring for grandchildren |  |  |
| Daily or Weekly | **-2.01±0.53***** | -0.13±0.38 |
| Monthly or Yearly | -1.09±0.64 | 0.23±0.41 |
| Caring for grandchildren× Time |  |  |
| Daily or Weekly | **0.38±0.18*** | -0.04±0.75 |
| Monthly or Yearly | 0.37±0.20 | 0.04±0.12 |

*** p<0.01, ** p<0.05, * p<0.10

^1^models for participants stratified by race. Model 1 : unadjusted; Model 2: were adjusted for age, sex, and poverty status .The main exposure variables HEI-2010, was from waves 3 and 4.

^2^ Boxes highlighted in green survived multiple testing

^Continuous covariates were centered at their mean.

#3: Time spent carding for grandchildren Daily|Weekly|Monthly|Yearly
