# Supplementary material for: Caregiver Status and Diet Quality in Community-Dwelling Adults
Source: Nutrients. 2021 May 26;13(6):1803. doi: 10.3390/nu13061803 (PMC8227086; doi:10.3390/nu13061803)
Supplement: Supplementary file 1 [file nutrients-13-01803-s001.zip › Supplementary table 2.docx]

**Supplementary table 2.** Longitudinal Associations of HEI-2010 components with caregiving questions 6^#^, stratified by race, for HANDLS participants (N=1,674) [β±SE, p-value]: Mixed-effects linear regression models^1^

|  | Whites (N=697) | African Americans (N=977) |
| --- | --- | --- |
| **Total Vegetables** |  |  |
| Time | 0.07±0.06 | 0.41±0.09 |
| Unpaid care for others |  |  |
| Daily or Weekly | -0.17±0.23 | -1.13±0.38 |
| Monthly or Yearly | -0.01±0.42 | 0.23±0.41 |
| Unpaid care for others× Time |  |  |
| Daily or Weekly | -0.05±0.10 | -0.04±0.11 |
| Monthly or Yearly | -0.03±0.20 | 0.04±0.12 |
| **Greens and Beans** |  |  |
| Time | -0.02±0.07 | 0.03±0.04 |
| Unpaid care for others |  |  |
| Daily or Weekly | 0.12±0.25 | 0.04±0.20 |
| Monthly or Yearly | -0.15±0.45 | 0.26±0.31 |
| Unpaid care for others× Time |  |  |
| Daily or Weekly | -0.16±0.11 | 0.07±0.06 |
| Monthly or Yearly | -0.20±0.22 | -0.008±0.10 |
| **Total Fruits** |  |  |
| Time | 0.12±0.06 | 0.03±0.04 |
| Unpaid care for others |  |  |
| Daily or Weekly | 0.10±0.28 | -0.23±0.21 |
| Monthly or Yearly | 0.07±0.51 | -0.21±0.32 |
| Unpaid care for others× Time |  |  |
| Daily or Weekly | -0.16±0.10 | -0.03±0.06 |
| Monthly or Yearly | 0.12±0.20 | 0.000±0.09 |
| **Whole Fruits** |  |  |
| Time | 0.12±0.06 | 0.06±0.04 |
| Unpaid care for others |  |  |
| Daily or Weekly | 0.31±0.28 | -0.22±0.19 |
| Monthly or Yearly | -0.14±0.50 | 0.04±0.29 |
| Unpaid care for others× Time |  |  |
| Daily or Weekly | -0.14±0.11 | -0.07±0.06 |
| Monthly or Yearly | 0.14±0.21 | -0.13±0.09 |
| **Whole grains** |  |  |
| Time | 0.03±0.09 | 0.03±0.05 |
| Unpaid care for others |  |  |
| Daily or Weekly | -0.03±0.41 | -0.10±0.30 |
| Monthly or Yearly | -0.13±0.74 | -0.46±0.46 |
| Unpaid care for others× Time |  |  |
| Daily or Weekly | 0.01±0.16 | -0.007±0.08 |
| Monthly or Yearly | -0.08±0.31 | -0.01±0.13 |
| **Total Dairy** |  |  |
| Time | -0.002±0.12 | 0.09±0.05 |
| Unpaid care for others |  |  |
| Daily or Weekly | -0.46±0.48 | 0.17±0.30 |
| Monthly or Yearly | 0.14±0.86 | -0.49±0.46 |
| Unpaid care for others× Time |  |  |
| Daily or Weekly | 0.04±0.22 | 0.07±0.08 |
| Monthly or Yearly | -0.02±0.41 | -0.14±0.14 |
| **Total Protein** |  |  |
| Time | 0.05±0.05 | -0.06±0.20 |
| Unpaid care for others |  |  |
| Daily or Weekly | 0.14±0.18 | 0.05±0.11 |
| Monthly or Yearly | 0.02±0.33 | 0.14±0.17 |
| Unpaid care for others× Time |  |  |
| Daily or Weekly | -0.05±0.08 | -0.003±0.03 |
| Monthly or Yearly | 0.11±0.16 | 0.04±0.05 |
| **Seafood and Plant Protein** |  |  |
| Time | 0.13±0.08 | -0.06±0.04 |
| Unpaid care for others |  |  |
| Daily or Weekly | -0.10±0.30 | 0.07±0.20 |
| Monthly or Yearly | -0.49±0.53 | -0.32±0.31 |
| Unpaid care for others× Time |  |  |
| Daily or Weekly | -0.03±0.13 | -0.003±0.06 |
| Monthly or Yearly | 0.18±0.25 | 0.04±0.10 |
| **Fatty Acid** |  |  |
| Time | 0.22±0.13 | -0.03±0.06 |
| Unpaid care for others |  |  |
| Daily or Weekly | -0.31±0.51 | 0.17±0.33 |
| Monthly or Yearly | -0.41±0.91 | 0.97±0.51 |
| Unpaid care for others× Time |  |  |
| Daily or Weekly | 0.22±0.23 | -0.04±0.01 |
| Monthly or Yearly | -0.25±0.43 | -0.14±0.17 |
| **Sodium** |  |  |
| Time | 0.03±0.11 | 0.06±0.06 |
| Unpaid care for others |  |  |
| Daily or Weekly | 0.30±0.53 | 0.08±0.34 |
| Monthly or Yearly | -0.03±0.96 | 0.03±0.52 |
| Unpaid care for others× Time |  |  |
| Daily or Weekly | 0.05±0.20 | -0.10±0.10 |
| Monthly or Yearly | 0.13±0.37 | -0.14±0.17 |
| **Refined Grain** |  |  |
| Time | 0.24±0.13 | 0.08±0.06 |
| Unpaid care for others |  |  |
| Daily or Weekly | 0.29±0.50 | -0.07±0.31 |
| Monthly or Yearly | -0.28±0.89 | 0.25±0.48 |
| Unpaid care for others× Time |  |  |
| Daily or Weekly | 0.05±0.22 | -0.08±0.10 |
| Monthly or Yearly | 0.65±0.42 | **-0.42±0.16***** |
| **Solid Fat and Added Sugar calories** |  |  |
| Time | 1.05±0.21 | 0.34±0.11 |
| Unpaid care for others |  |  |
| Daily or Weekly | -1.81±0.97 | -0.68±0.61 |
| Monthly or Yearly | -0.28±1.75 | 1.10±0.94 |
| Unpaid care for others× Time |  |  |
| Daily or Weekly | 0.32±0.37 | 0.20±0.17 |
| Monthly or Yearly | -0.14±0.70 | -0.18±0.29 |

*** p<0.01, ** p<0.05, * p<0.10

^1^models for participants stratified by race. Model 1 : unadjusted; Model 2: were adjusted for age, sex, and poverty status .The main exposure variables HEI-2010, was from waves 3 and 4.

^Continuous covariates were centered at their mean.

#6: Time spent carding for others, not children and grandchildren Daily|Weekly|Monthly|Yearly

** Boxes highlighted in green survived correction for multiple testing
